# Seasonal variation in C:N:P stoichiometry, nonstructural carbohydrates, and carbon isotopes of two coniferous pioneer tree species in subtropical China

**DOI:** 10.3389/fpls.2023.1225436

**Published:** 2023-11-27

**Authors:** Yuanxi Liu, Jiandong Xiao, Jianli Sun, Zhijuan Zhao, Xin Deng, Junwen Wu, Deguo Zhang, Yun Bao

**Affiliations:** ^1^ College of Forestry, Southwest Forestry University, Kunming, Yunnan, China; ^2^ Yunnan Academy of Ecological and Environmental Sciences, Kunming, Yunnan, China; ^3^ College of art and design, Southwest Forestry University, Kunming, Yunnan, China

**Keywords:** pioneer tree species, winter and spring drought, needle carbon stable isotope, stoichiometry, nonstructural carbohydrates, seasonal variation

## Abstract

The characteristics of C:N:P stoichiometry, nonstructural carbohydrate (NSC) content, and C stable isotopes and their relationships affect plant responses to environmental changes and are critical to understanding the ecosystem carbon and water cycles. We investigated the water use strategies and physiological changes of two pioneer tree species (*Pinus armandii* and *Pinus yunnanensis*) in response to seasonal drought in subtropical China. The seasonal variation in needle δ^13^C values, C:N:P stoichiometry, and NSC contents of the two tree species were studied in 25-year-old plantation in central Yunnan Province. The needle δ^13^C values of both species were highest in summer. Soluble sugars, starch and NSC content of the two tree species decreased from spring to winter, while there was no significant difference in the seasonal variation of soluble sugars/starch in *P. armandii* needles, the maximum soluble sugars/starch in *P. yunnanensis* needles was in autumn. In addition, the C, N, and P contents of the needles and the C:N and C:P ratios of the two species showed different seasonal fluctuations, whereas the N:P ratio decreased with the season. The C:N:P stoichiometry and NSC content of the needles showed significant correlations, whereas the needle δ^13^C was weakly correlated with C:N:P stoichiometry and NSC content. Phenotypic plasticity analysis and principal component analysis revealed that the needle nutrient characteristics (NSC and P contents and N:P ratio) and needle δ^13^C values were critical indicators of physiological adaptation strategies of *P. armandii* and *P. yunnanensis* for coping with seasonal variation. These results increase our understanding of the water-use characteristics of the two pioneer tree species and the dynamic balance between the NSC, C, N, and P contents of the needles.

## 1 Introduction

Carbohydrates are the main products of plant photosynthesis and can be divided into structural carbohydrates (SC) and nonstructural carbohydrates (NSC) (Martínez-Vilalta et al., 2016). NSC are composed of soluble sugars and starches and provide energy for plant growth and metabolism. Soluble sugars are required for plant metabolism, carbohydrate transport, and utilization. Their osmoregulatory functions ensure plant growth and development in different environments (Blum, 2017). In contrast, starches are not directly involved in plant growth but represent a critical source of soluble sugars, which are converted to starch and vice versa under specific conditions, regulating the relationship between carbon supply and demand and altering the carbon metabolism levels (Furze et al., 2019). Therefore, analyzing changes in the NSC content in plant tissues helps to understand the carbon balance and the physiological response and adaptation of plants to the environment. NSC reserves and allocation in plant organs and tissues are influenced by light (Gansert and Sprick, 1998), temperature (Sauter, 1988), climate (McDowell et al., 2008; Woodruff and Meinzer, 2011), ozone (Battistelli et al., 2001), altitude (Bernoulli and Körner, 1999), and tree age and species (Cerasoli et al., 2004; Woodruff and Meinzer, 2011; Liu et al., 2023). NSC stored in plant tissues are critical under unfavorable environmental conditions. For example, many tree species mobilize stored NSC during the drought season to meet the metabolic demands of plants (Ryan, 2011). Therefore, drought reduces NSC in plants (Sala et al., 2010), resulting in carbon depletion and ultimately increased tree mortality (McDowell et al., 2008). A study by Oleksyn et al. (2000) on the seasonal variation of the NSC content in European red pine (*Pinus sylvestris*) showed that starch accumulation in needles began when the daily minimum temperature was consistently above 0°C and reached a peak before leaf emergence. The maximum starch accumulation occurred in annual needles; it declined in late spring and reached a minimum in early summer. The soluble sugar content of the needles was lowest in late spring to summer and higher in autumn and winter to withstand low-temperature stress. Würth et al. (2005) investigated 17 tropical rainforest tree species and found that the leaf NSC content was significantly higher in the dry season than in the rainy season because drought stress inhibits tree growth.

Carbon (C), nitrogen (N), and phosphorus (P) are three major elements essential for plant growth and adaptation to terrestrial habitats, C provides the structural basis for plants, and N and P are essential nutrients critical for primary production (Cao and Chen, 2017). Leaf C, N, and P contents are associated with many key functions of plant growth and reproduction and can be used as indicators to evaluate plant nutrient utilization and the response to environmental changes (Qin et al., 2019). Leaf stoichiometry is an indicator of the genetic characteristics of plants and their adaptation to environmental conditions. Therefore, the study of plant leaf stoichiometry can improve our understanding of biogeochemical cycles and ecosystem structure and function (Bai et al., 2020). It has been shown that the leaf C, N, and P contents increase with elevation (Han et al., 2005; Sardans and Peñuelas, 2008); however, studies of tropical and subtropical mountains have shown the opposite trend (Gessler et al., 2017). Changes in the leaf nutrient content and stoichiometry are primarily attributed to climate, soil type, and ecosystem development (Ke et al., 2014). Seasonal variation had a significant impact on leaf C, N, and P stoichiometry of *Quercus suber* L. (Orgeas et al., 2003) and *Phragmites australis* in Dunhuang (Liu et al., 2020). There is evidence that climate (mean annual temperature (MAT) and mean annual precipitation (MAP)) are the dominant factors regulating plant C:P and N:P ratios (Zhang et al., 2018). During the dry season, plants conserve water by increasing the N and P input into non-photosynthetic tissues or organs to increase cellular osmotic pressure, an effective strategy for plants to cope with drought conditions (Gessler et al., 2017).

The stable carbon isotope composition (δ^13^C) of leaves is a reliable indicator of long-term water-use efficiency (WUE) (Shen et al., 2017) and a useful proxy for studying long-term WUE in plants (Farquhar et al., 1989). It has been widely used for the analyses of plant leaves (Wang et al., 2008; Jiang et al., 2020), canopies (Medrano et al., 2015), communities (Durand et al., 2020) and ecosystems (Zhu et al., 2015). Most studies have focused on the leaf scale (Schäfer et al., 2018) because it reveals the water-use mechanisms of plants and is the basis for larger-scale water-use studies that consider the effects of climatic and physiological factors on plant carbon assimilation and stomatal conductance (Farquhar et al., 1989). Farquhar et al. (1989) observed a significant positive correlation between leaf δ^13^C values and WUE of C3 plants. High δ^13^C values of plant leaves in the same habitat usually indicate high WUE and drought resistance (Brienen et al., 2017). In arid and semi-arid regions, plant leaf δ^13^C values tend to increase as plant water availability decreases, indicating conservative water use (Wang et al., 2020; Imin et al., 2021).

Many studies have been conducted on the seasonal variation of plant NSC content, C:N:P stoichiometry, and leaf δ^13^C values. For example, the leaf N content was positively correlated with the NSC content, and P has been identified as a key element in plant metabolism (McGroddy et al., 2004). Analysis of plant leaves in the subtropics by Huang et al. (2015) showed that plant WUE was not significantly correlated with leaf nitrogen concentration in a phosphorus-limited context, while it was significantly and positively correlated with leaf phosphorus concentration. Cernusak et al. (2010) investigated the leaves of tropical tree species and found a positive correlation between the N/P ratio and WUE. However, few studies have examined the effects of seasonal variation on the NSC content, C:N:P stoichiometry, and δ^13^C values of plants. Therefore, we investigated the effects of the NSC content, C:N:P stoichiometry, and needle δ^13^C values of *Pinus yunnanensis* and *Pinus armandii* needles in different seasons. Both species are important pioneer species in the subtropical region of southwest Yunnan Province, China. The purpose of this study was to understand needle nutrients and plant water use in different seasons to improve plantation productivity. The objectives were to investigate the seasonal variations in needle δ^13^C values, the NSC, C, N, and P contents, and the C:N:P ratio of *P. armandii* and *P. yunnanensis*. This study aims to address the following questions: 1) whether the needle δ^13^C, NSC and C, N, and P content of seasonal variation is similar for two pioneer tree species (*P. armandii* and *P. yunnanensis*.)? 2) The relationship between needle δ^13^C values, NSC and C:N:P stoichiometry of two pioneer tree species.

## 2 Materials and methods

### 2.1 Study area

The experimental site is located in a state-owned forest area in Yiliang County, Kunming City, Yunnan Province (26°11′-26°25′N, 101°27′-101°28′E) at an altitude of 1300-2800 m above sea level. The area has a subtropical monsoon climate, with dry conditions in winter, little precipitation in spring, wet summers, and mild winters. The rainy season lasts from May to October, accounting for about 85% of the annual precipitation, and the dry season is from November to April, accounting for about 15% of the annual precipitation. The average annual precipitation is 912.2 mm, the average annual temperature is 16.3°C, the number of sunshine hours is 2,177.3 hours, and the frost-free period is 260 days.

### 2.2 Experimental setup

P. *yunnanensis* and *P. armandii* woodlands with uniform vegetation and strong regional representation were selected as sample plots (**Table 1**). Three standard plots (30 × 30 m) were randomly chosen, and the distance between the plots was greater than 30 m. The plots were located on upper and middle slopes with an average elevation of 2343 m. Six trees (three *P. armandii* and three *P. yunnanensis*) were selected in medium-aged stands in each plot, 3 sample plots, a total of nine *P. yunnanensis* and nine *P. armandii* were selected from the three sample plots. They had an average diameter at breast height of 20.44 cm and an average height of 12.59 m. The soil type was red loam with 27.1 g·kg^-1^ organic carbon, 1.27 g·kg^-1^ total nitrogen, and 0.57 g·kg^-1^ total phosphorus.

**Table 1:** Plot information.

Sample Site	Elevation(m)	Longitude and latitude	Slope position and direction
1	2346.0	24˚54’4”N 103˚5’9”E	Mid-slope, south
2	2319.0	24˚53’48”N 103˚5’9”E	Mid-slope, east
3	2335.0	24˚53’48”N 103˚5’37”E	Mid-slope, south of west

### 2.3 Tree sampling

We obtained samples on January 20 (spring), May 29 (summer), August 1 (fall), and November 9 (winter) of 2021. Needles from the middle layer of the canopy were selected and represented the needles of the entire canopy. We collected three first-degree branches (first-degree branches were connected to the trunk, second-degree branches were connected to first-degree branches, and third-degree branches were connected to second-degree branches) from each sample tree to eliminate sampling errors in needle nutrient composition due to differences in branch orientation. Three tertiary branches were randomly cut with high branch shears on selected primary branches of each sample tree in four directions. They were evenly mixed, and the needles of each age group were separated by branch. One mixed needle sample was taken from each of the three sample trees in each plot. The needle samples were bagged and used to determine needle δ^13^C values and the C, N, and P contents. The samples were transported to the laboratory, rinsed, and placed in an oven at 105°C to prevent enzymatic carbohydrate reactions. They were subsequently dried at 80°C to a constant dry weight, crushed in a pulverizer, and stored in a sealed container.

### 2.4 Analysis methods

#### 2.4.1 Determination of NSC, C, N, and P contents of needle

The soluble sugars and starch contents of the *P. yunnanensis* needles were measured using the phenol-sulfuric acid colorimetric method (Yang and Luo, 2011). The NSC content in the needles was the sum of the soluble sugar and starch contents. The total C content was determined by the potassium dichromate method plus dilution heating, the total N content was determined by the colorimetric method, and the total P content was determined by the molybdenum-antimony anti-colorimetric method. The results were expressed as g·kg^-1^.

#### 2.4.2 Analysis of needle δ^13^ values

Needles δ^13^C values were determined with a stable isotope ratio mass spectrometer (DELTA V; EA-HT (Elemental Analyzer); Bremen) with an error of less than 0.15 ‰.

Principle: The sample was burnt at a high temperature in the elemental analyzer to produce CO_2_, and a mass spectrometer was used to calculate the δ^13^C value of the sample by detecting the ^13^C to ^12^C ratio of CO_2_ and comparing it with an international standard (Pee Dee Belnite or PDB). It was calculated as follows (Farquhar et al., 1989):


(1) $$\delta^{13}C=\;\left(R_{sam}/R_{std}-1\right)\;\times \;1000$$

where δ^13^C is the carbon isotope value of the sample, R_sam_ and R_std_ are the ratios of the heavy and light isotopic abundances of the elements in the sample and the international standard, respectively (^13^C/^12^C).

Determination accuracy: δ^13^C: ±<0.1 ‰ (non-labeled samples).

#### 2.4.3 Statistical analyses

All values (δ^13^C values and the NSC (soluble sugars and starch), C, N, and P contents of the *P. armandii* and *P. yunnanensis* needles) were expressed as mean ± standard deviation (n=9). The meteorological data of the sample sites were obtained from the National Meteorological Science Data Center (http://www.cma.gov.cn). SPSS 26.0 software was used to conduct ANOVA (One-way ANOVA), calculate the least square differences (LSD), and perform multiple comparisons (*P* = 0.05) of the data. Pearson’s correlation analysis was conducted. The significance levels were highly significant (*P*<0.01) and significant (*P*<0.05). Plasticity index: P=(X_max_-X_min_)/X_max_, where X_max_ and X_min_ denote the maximum and minimum value of each indicator. Graphpad prism 8.0 and Origin 9.0 software were used to create graphs, and the data in the graphs were expressed as the mean ± the standard error.

## 3 Results

### 3.1 Needle δ^13^C values

**Figure 1:**
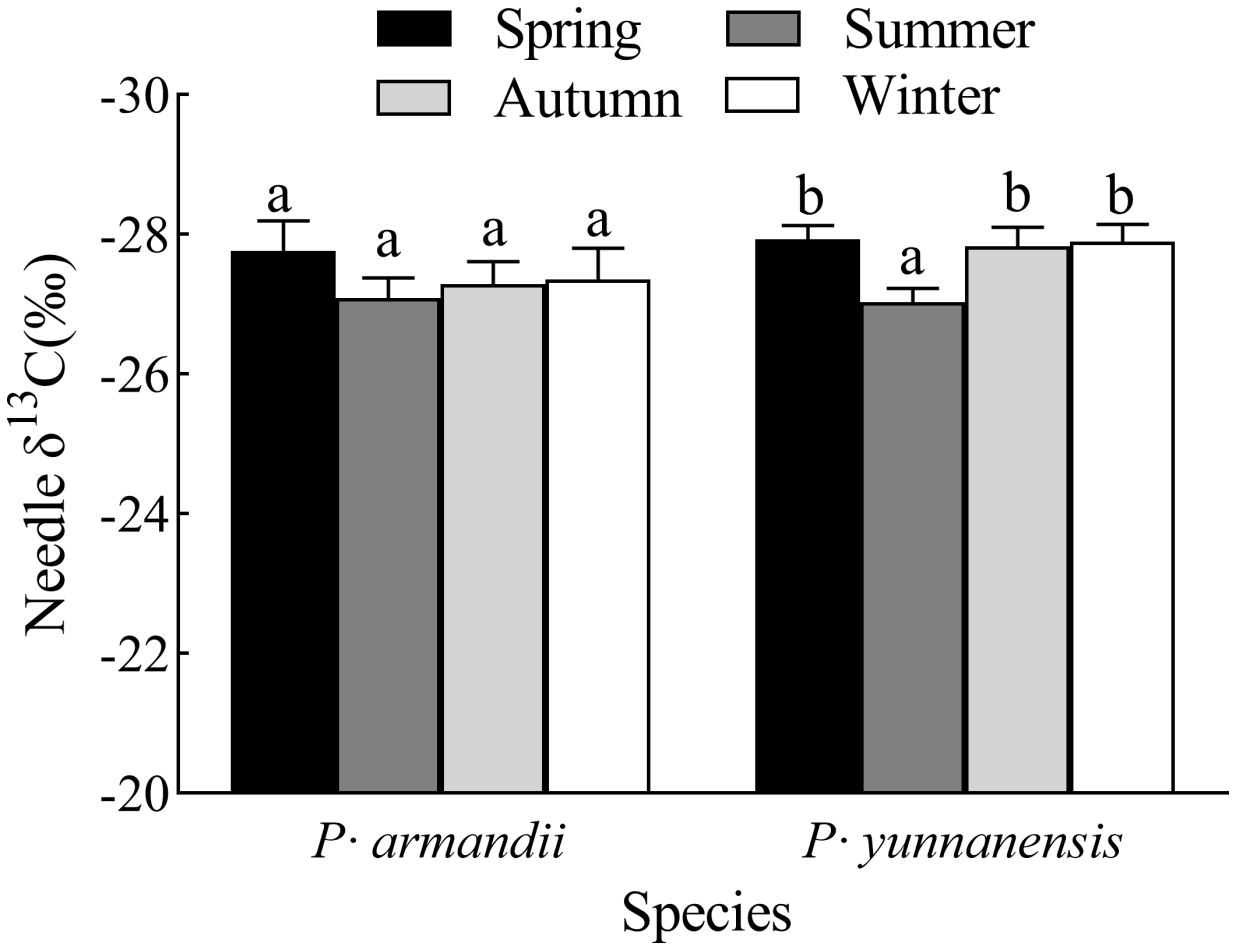
Seasonal variation of δ^13^C values of the needles of two tree species. Th error bars indicate the standard deviation of the mean (n=9). Different letters indicate significant differences between seasons (*P*< 0.05).

The seasonal variation of the needle δ^13^C values was similar for the two species (**Figure 1**). There was no significant difference (*P* > 0.05) in the δ^13^C values of *P. armandii* needles between the four seasons. They were highest in summer (-27.09‰) and lowest in spring (-27.77‰). In contrast, the δ^13^C values of the *P. yunnanensis* needles were significantly higher in summer (-27.03‰) than in the other seasons (*P*< 0.05).

### 3.2 Needle NSC content

**Figure 2:**
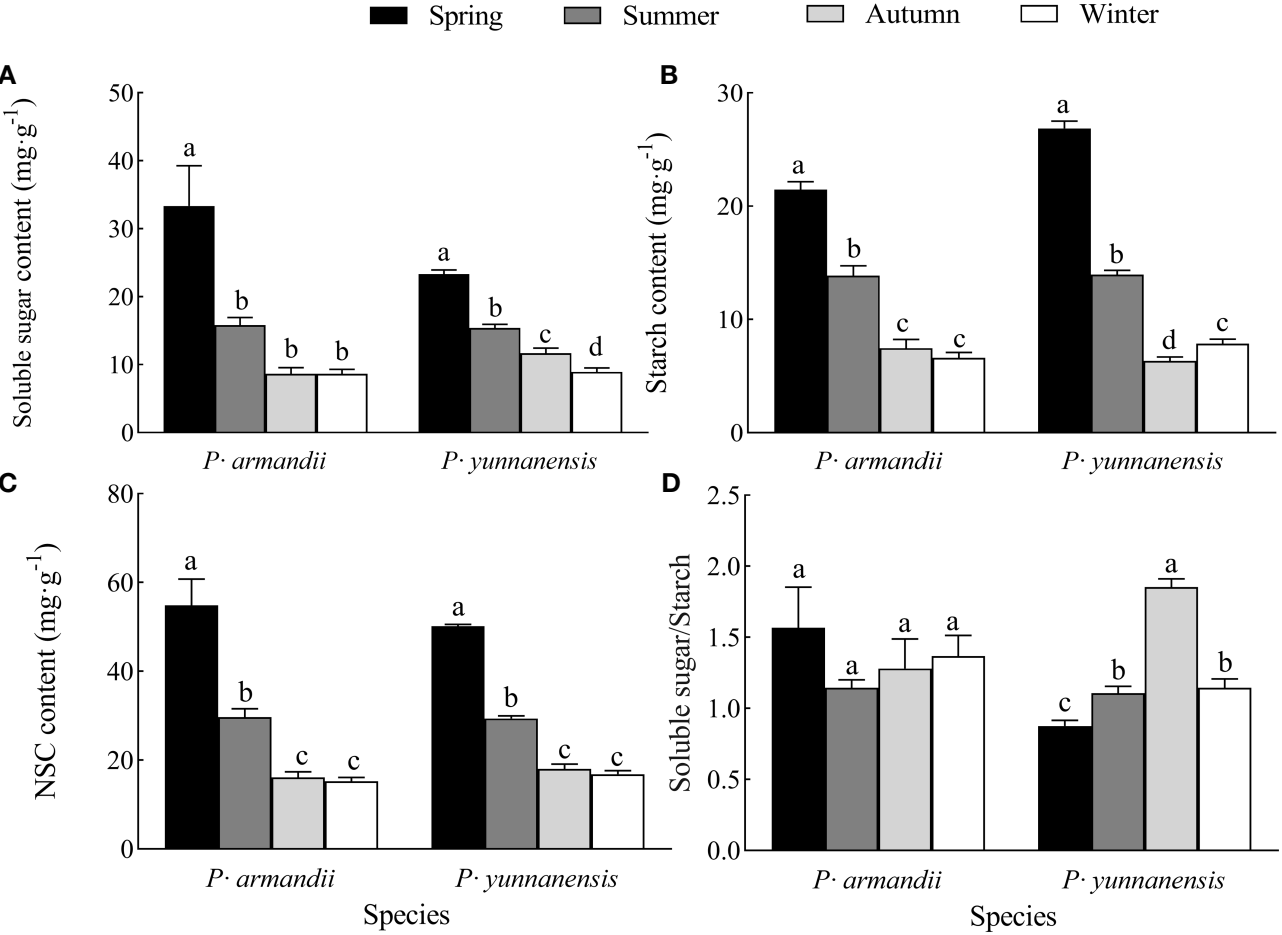
Seasonal variation of soluble sugar **(A)**, starch **(B)**, and NSC **(C)** contents of needles and their soluble sugar/starch **(D)** ratio in two tree species.

The soluble sugar, starch, and NSC contents of the needles (the sum of soluble sugar and starch concentrations) of both species decreased at different rates seasonally, and the highest values occurred in spring (**Figure 2**). The season significantly affected (*P*<0.05) the needles’ soluble sugar, starch, and NSC contents in *P. yunnanensis* and *P. armandii*. In *P. armandii*, the soluble sugar, starch, and NSC contents decreased significantly (*P*<0.05) from spring to summer and from summer to autumn and maintained a steady decrease from autumn to winter (*P*>0.05). In *P. yunnanensis*, the soluble sugar content decreased from spring to winter (**Figure 2A**), the starch content decreased from spring to autumn and increased in winter (**Figure 2B**), and the NSC content decreased from spring to autumn and then remained in winter (**Figure 2C**).

The leaf soluble sugar/starch ratio reflects the distribution of NSC in the leaves and provides insights into the nutrient utilization of plants. The soluble sugar/starch ratio of the *P. armandii* needles varied seasonally and remained stable (**Figure 2D**), whereas that of the *P. yunnanensis* needles increased and decreased, reaching a maximum (1.853) in autumn (**Figure 2D**).

### 3.3 Needle C:N:P stoichiometry

**Figure 3:**
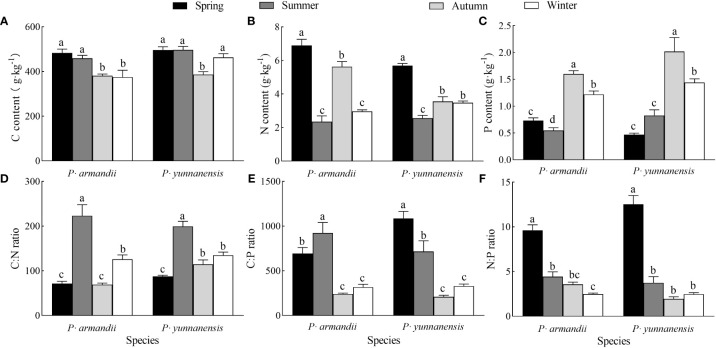
Seasonal variation in C content **(A)**, N content **(B)**, P content **(C)**, C:N ratio **(D)**, N:P ratio **(E)**, C:P ratio **(F)** of needles of two tree species.

There was a significant difference (*P*<0.05) in the needle C, N, and P contents of the two species in different seasons (**Figure 3**). The needle C content of *P. armandii* decreased over the seasons, whereas that of *P. yunnanensis* decreased and increased, reaching the lowest value (386.100 g·kg^-1^) in autumn (**Figure 3A**). The N content of the *P. armandii* needles decreased, increased, and decreased over the seasons, whereas that of the *P. yunnanensis* needles decreased and increased, reaching the minimum value (2.559 g·kg^-1^) in summer (**Figure 3B**). The P content of the *P. armandii* needles decreased, increased, and then decreased over the seasons, reaching the maximum value in autumn (1.602 g·kg^-1^). In contrast, the N content of the *P. yunnanensis* needles increased and decreased, reaching the minimum value in autumn (2.019 g·kg^-1^) (**Figure 3C**).

The C:N:P ratios of the two species differed significantly (*P*<0.05) in different seasons (**Figure 3**). The needle C:N ratios of *P. armandii* and *P. yunnanensis* increased, decreased, and increased over the seasons, and both reached the maximum values in summer (223.070 for *P. armandii* and 199.396 for *P. yunnanensis*) (**Figure 3D**). The C:P ratio of the *P. armandii* needles increased and decreased over the seasons, reaching the maximum value in summer (923.774). In contrast, the C:P ratio of the *P. yunnanensis* needles decreased, reaching the maximum value in spring (1086.716) (**Figure 3E**). The N:P ratios of the needles of *P. armandii* and *P. yunnanensis* decreased over the seasons, and both reached the maximum values in spring (9.624 for Pinus sylvestris and 12.535 for *P. yunnanensis*) (**Figure 3F**).

### 3.4 Correlation between needle δ^13^C values, C:N:P stoichiometry, and NSC content

**Figure 4:**
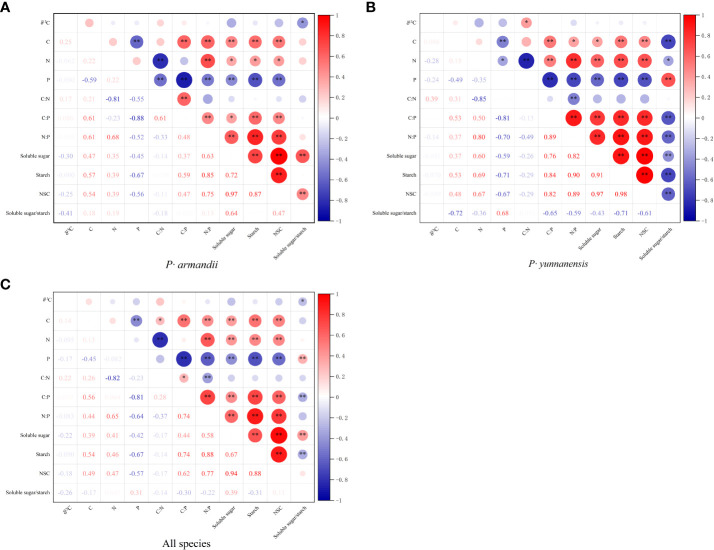
Correlation coefficients between the needle δ^13^C values, C:N:P stoichiometry, and NSC content of two tree species. **(A)** *P· armandii*; **(B)** *P· yunnanensis*; **(C)** both species (disregard for species differences). *P< 0.05, **P< 0.01.

There was a significant correlation between the C:N:P stoichiometry and the NSC content of the *P. yunnanensis* and *P. armandii* needles, whereas the correlation between the δ^13^C values, C:N:P stoichiometry, and NSC was weak (**Figure 4**). As shown in **Figure 4A**, the needle δ^13^C values of *P. armandii* were negatively correlated only with the soluble sugar/starch ratio. The needle C content was negatively correlated with the P content and positively correlated with the C:P and N:P ratios and the contents of soluble sugars, starch, and NSC. The needle N content was negatively correlated with the C:N ratio and positively correlated with the N:P ratio and the soluble sugar, starch, and NSC contents. The needle P content was negatively correlated with the C:N and N:P ratios, and the soluble sugar, starch, and NSC contents.

As shown in **Figure 4B**, the δ^13^C values of the *P. yunnanensis* needles were positively correlated only with the C:N ratio. The needle C content was negatively correlated with the P content and the soluble sugar/starch ratio and positively correlated with the C:P and N:P ratios and the soluble sugar, starch, and NSC contents. The needle N content was negatively correlated with the P content, C:N ratio, and soluble sugar/starch ratio and positively correlated with the C:P and N:P ratios and the soluble sugar, starch, and NSC contents. The P content was negatively correlated with the C:P and N:P ratios and the soluble sugar, starch, and NSC contents and positively correlated with the soluble sugar/starch ratio.

**Figure 4C** shows the correlations for both tree species. The needle δ^13^C values were negatively correlated only with the soluble sugar/starch ratio. The needle C content was negatively correlated with the P content and the soluble sugar/starch ratio and positively correlated with the C:N, C:P, and N:P ratios and the soluble sugar, starch, and NSC contents. The needle N content was negatively correlated with the C:N ratio and positively correlated with the N:P ratio and the soluble sugar, starch, and NSC content. Needle P content was positively correlated with the C:N, C:P, and N:P ratios and the soluble sugar, starch, and NSC content.

### 3.5 Phenotypic plasticity index of needle δ^13^C, C:N:P stoichiometry, and NSC content

**Figure 5:**
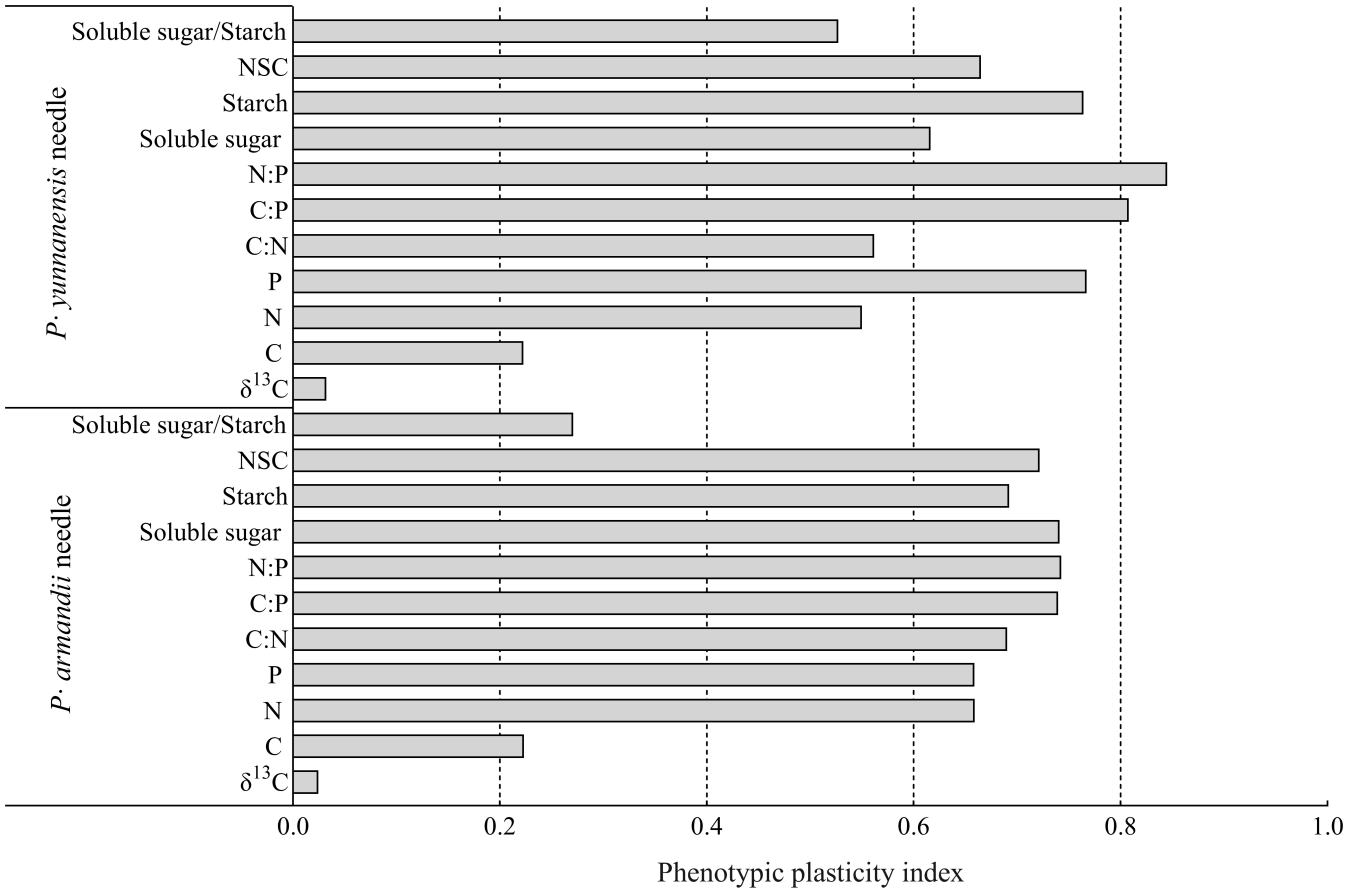
Phenotypic plasticity indices of *P. armandii* and *P. yunnanensis* needle δ^13^C, C:N:P stoichiometry, and NSC content.

The phenotypic plasticity index was calculated for the physiological and biochemical parameters of the needles of *P. armandii* and *P. yunnanensis* (**Figure 5**). The needle δ^13^C had the lowest plasticity indices (0.024 for *P. armandii* and 0.032 for *P. yunnanensis*), and the N:P ratio had the largest plasticity indices (0.743 for *P. armandii* and 0.845 for *P. yunnanensis*). Among the indicators of *P. armandii* needles, needle C/P (0.740), N/P (0.743), Soluble sugar (0.741) and NSC (0.722) were the four indicators of plasticity with large variations. Among the indicators of *P. yunnanensis* needles, needle N/P (0.845), C/P (0.808), P (0.767) and Starch (0.764) were the four indicators with greater d variation in plasticity. The phenotypic plasticity indices of the *P. yunnanensis* parameters needles were higher than those of the *P. armandii* parameters.

### 3.6 Principal component analysis of δ^13^C, C:N:P stoichiometry, and NSC content

**Figure 6:**
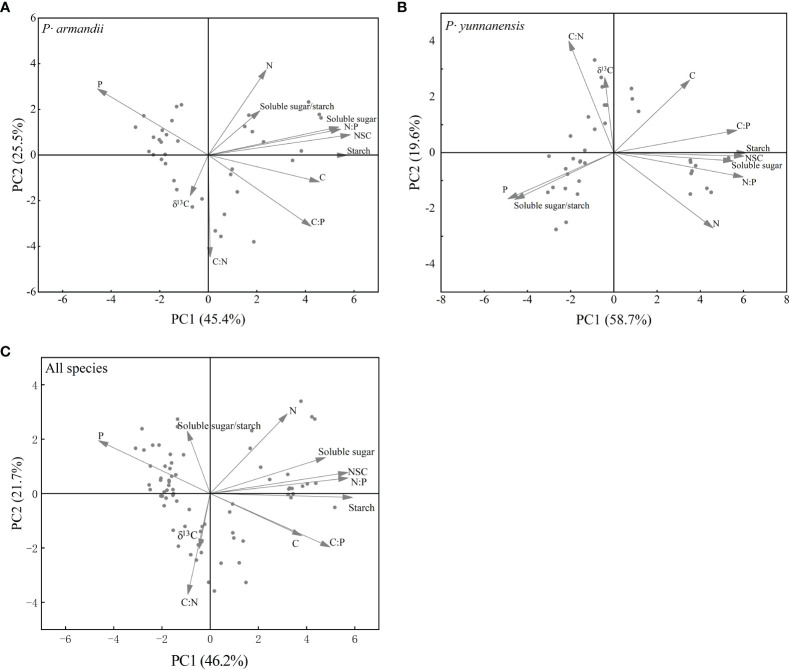
Principal component analysis (PCA) of δ^13^C and the physiological and biochemical parameters of *P. armandii* and *P. yunnanensis* needles in summer, autumn, winter, and spring in 2021/2022. Needle δ^13^C, C:N:P stoichiometry, soluble sugar, starch, and NSC contents, and soluble sugar/starch ratio. **(A)** *P· armandii*; **(B)** *P· yunnanensis*; **(C)** both species (disregard for species differences).

**Figure 6** shows the results of the principal component analysis. Differences were observed in the seasonal variation of the physiological indicators of *P. armandii* and *P. yunnanensis*. The cumulative variance contributions of the first two principal components for *P. armandii* and *P. yunnanensis* were 70.9% (**Figure 6A**; axis 1 = 45.4% and axis 2 = 25.5%) and 78.3% (**Figure 6B**; axis 1 = 58.7% and axis 2 = 19.6%), respectively. The ranking of the physiological indicators of the *P. armandii* needles was the starch content, NSC content, N:P ratio (positive axis), and P content (negative axis) on the first axis, and the N content (positive axis) and C:N ratio (negative axis) on the second axis. The ranking of the physiological indicators of the *P. yunnanensis* needles was the starch content, NSC content, soluble sugar content, N:P ratio (positive axis), and the P content on the first axis (negative axis), and in the C:N ratio (positive axis) and N content (negative axis) on the second axis. The cumulative variance contributions of the first two principal components of the two species were 67.9% (**Figure 6C**; axis 1 = 46.2% and axis 2 = 21.7%). The ranking of the physiological indicators of the two species was the starch content, NSC content, N:P ratio (positive axis), and P content on the first axis (negative axis) and the N content (positive axis) and C:N ratio on the second axis (negative axis).

## 4 Discussion

### 4.1 Seasonal variation of needle δ^13^C values

Leaf δ^13^C is a reliable indicator of the long-term WUE of plants (Farquhar et al., 1989; Shen et al., 2017). Temperature and water availability are the primary factors affecting plant WUE, and the latter is the most significant factor (Liu, 1998). Plants adapt their physiological functions to different water conditions. When plant growth is constrained by water conditions, plants reduce water transpiration and stomatal conductance to maintain growth (Wu et al., 2017). The needle δ^13^C values of *P. armandii* and *P. yunnanensis* reached the maximum in summer (**Figure 1**). This season is the maximum growth period of needles, and the plants have the highest demand. Drought stress requires adaptation, resulting in the high WUE of both species (**Table 1**). This finding indicates that precipitation substantially affected the WUE of the two tree species experiencing drought stress. Our results are consistent with those of Garten et al. (1992) for *Quercus prinus* and Nie et al. (2014) for *Radermachera sinica*, *Sapium rotundifolium*, *Sterculia euosma*, *Schefflera octophylla*, *Alchorena trewioides*, and *Vitex negundo*. The plants responded to a dry environment by increasing the leaf δ^13^C values (higher WUE). The seasonal differences can be attributed to the plants’ water use strategy, shifting from regular water use in the rainy season to conservative water use in the dry season, depending on seasonal precipitation. However, the phenotypic plasticity index analysis showed that the δ^13^C values of the needles of both two tree species (**Figure 5**) were the smallest. And the principal component analysis (**Figure 6**) also showed low loading values. These two results again explain the low sensitivity of the two species’ plant needle δ13C values to seasonal changes, and one of the main strategies by which the two species have a strong fitness for use as pioneer tree species for the restoration and improvement of fragile karst habitats in subtropical China.

### 4.2 Seasonal variation of needle NSC content

NSC represent an energy source for the growth and metabolism of plants (O’Brien et al., 2014; Hartmann and Trumbore, 2016; Liu et al., 2020). Soluble sugars are photosynthetic products required for plant growth, development, and osmoregulation (Dietze et al., 2014). Starch is stored to meet the plant’s energy needs (Hartmann and Trumbore, 2016). The NSC content reflects the relationship between stored C (photosynthesis) and plant respiration and growth (Richardson et al., 2015; Yang et al., 2016).

In this study, the soluble sugar, starch, and NSC contents of *P. armandii* and *P. yunnanensis* needles decreased at different rates over the seasons (**Figure 2**). Moreover, the soluble sugar, starch, and NSC contents of *P. armandii* and *P. yunnanensis* needles were important indicators of phenotypic plasticity, and the degree of change in the content reflected the degree of the species’ response to seasonal variation. The plants had to use soluble sugars and NSC for growth and development under drought conditions, resulting in an increase in the soluble sugar and NSC contents in the needles (Ayub et al., 2011; Ramírez-Briones et al., 2017). The starch in the needles had accumulated in the previous year due to the sprouting of new leaves, causing the highest needle starch content in *P. yunnanensis* in January. As the growing season progressed, the stored carbohydrates were utilized (soluble sugars in needles and starch decomposition into soluble sugars) during protoplast extension. The needles synthesized small amounts of soluble sugars for their growth. Thus, the carbon assimilation rate was lower than the carbon consumption rate, and the NSC content decreased. This result agrees with those of Mei et al. (2015) for Xing’an larch (*Larix gmelinii*) and ash (*Fraxinus chinensis*). However, in our study, the NSC content (including the soluble sugar and starch contents) did not increase during the late growing season, this may be an internal regulatory mechanism in plants.

The soluble sugar/starch ratio in plants reflects the mutual conversion of soluble sugar and starch in response to environmental changes (Li et al., 2008; Han et al., 2020). In this study, the soluble sugar/starch ratio of *P. armandii* needles varied seasonally and remained stable, whereas that of *P. yunnanensis* needles increased and decreased, reaching a maximum in autumn (**Figure 2**). The phenotypic plasticity index of the soluble sugar/starch ratio was higher for *P. yunnanensis* than for *P. armandii* (**Figure 5**). The principal component loading value of the soluble sugar/starch ratio was higher for *P. yunnanensis* than for *P. armandii* (**Figure 6**). These findings indicated that *P. yunnanensis* responded to seasonal variation by regulating the conversion of needle soluble sugar and starch, whereas *P. armandii* did not. The results of the phenotypic plasticity analysis (**Figure 5**) and principal component analysis (**Figure 6**) demonstrated that the needle starch content (storage of starch and conversion to soluble sugars) was an indicator of the physiological strategies of both tree species to cope with seasonal variation.

### 4.3 Seasonal variation of C:N:P stoichiometry of needle

Ecological stoichiometry is used to analyze plant energy use efficiency and their ability to maintain nutrient levels and produce photosynthetic products (Zhang et al., 2018). Plant leaves absorb carbon dioxide through photosynthesis, which requires enzyme (N) catalysis. Enzyme synthesis requires the replication of RNA (P), reflecting the coupling between the three elements C, N, and P (Domingues et al., 2010; He et al., 2019). The mean C content of the needles of *P. yunnanensis* in young and medium-aged stands in this study (**Figure 3**) was close to the global leaf C content of terrestrial plants (464 g-kg-1) (Elser et al., 2000). The needle N and P contents (**Figure 3**) were lower than the global-scale N and P contents (20.60 g-kg-1, 1.99 g-kg-1) (Elser et al., 2000) and the national average of 753 terrestrial plant species (18.6 g-kg-1, 1.21 g-kg-1) (Han et al., 2005). The C:N:P stoichiometric ratios of *P. armandii* and *Pinus yunnanensis* needles in this study showed seasonal dynamics. The C content of *P. armandii* needles decreased over the seasons, and that of *P. yunnanensis* needles was lowest in autumn (**Figure 3A**). During the rapid growth phase of the tree needles, the biomass increases rapidly, reducing the C content (Sardans and Peñuelas, 2008). The ranking of the N contents of *P. armandii* and *P. yunnanensis* was spring > autumn > winter > summer (**Figure 1B**). Thus, more N is stored in the plants, and when nutrient uptake occurs during drought, a higher nutrient level increases the N content. The nutrients are used for the growth of non-photosynthetic tissues or organs, increasing cellular osmotic pressure and conserving water (Gessler et al., 2017). The P content of *P. armandii* and *P. yunnanensis* needles was higher in autumn and winter (**Figure 3C**) and reached the minimum value in summer, in agreement with Ågren (2008), who observed a higher P concentration in plant tissues during rapid growth periods.

The leaf C/N and C/P ratios are critical physiological indicators of the plant growth rate and carbon assimilation capacity (Wang et al., 2018). The leaf C/N and C/P ratios are inversely correlated with the N and P use efficiencies, i.e., the amount of total organic matter lost or stored per unit of nutrients (Vitousek, 1982). Leaf C/N and C/P ratios are important physiological indicators related to plant growth rate and plant carbon assimilation capacity (Wang et al., 2018); they can also reflect the efficiency of plant use of N and P nutrients. Leaf C/N and C/P are inversely proportional to the efficiency of N and P use by the plant, i.e. the amount of total organic matter lost or stored per unit of nutrients (Vitousek, 1982). In this study, the C:N ratios of *P. armandii* and *P. yunnanensis* needles were highest in summer (**Figure 3D**), whereas the C:P ratio was higher in spring and summer than in the other seasons (**Figure 3E**). This result indicates that both species had the highest N use efficiency during summer and the highest P use efficiency and assimilation ability during spring and summer. The leaf N/P ratio is an indicator of N saturation and nutrient limitations (Wang et al., 2018). Rong et al. (2015) found that plant growth was limited by P when the N/P ratio exceeded 16 and limited by N when the N/P ratio was less than 14. In this study, the N:P ratios of the needles of the two tree species decreased over the seasons and were less than 14 (**Figure 3F**), indicating that growth was limited by N. Both tree species had larger phenotypic plasticity indices (**Figure 5**) and larger principal component loadings (**Figure 6**), indicating this indicator of needle N:P ratio is important for seasonal variation in both tree species. An analysis of the seasonal dynamics of the ratios is critical to understanding nutrient storage and the long-term productivity of the two tree species.

### 4.4 Relationships between needle δ^13^C values, NSC content, and C:N:P stoichiometry in two tree species

The nutrient status (N and P) of trees affects WUE by influencing the photosynthetic rate or stomatal conductance. The leaf N concentration is positively correlated with the photosynthetic CO_2_ assimilation rate as N deposition increases (Evans, 1989). Jacob and Lawlor (1991) showed that leaf conductance affected the photosynthetic rate more than stomatal conductance when phosphorus shortage occurred. Talbi-Zribi et al. (2011) found a positive correlation between leaf phosphorus concentration and photosynthetic rate. Garrish et al. (2010) observed that plant WUE was related to soil N availability but not soil phosphorus availability. In contrast, Huang et al. (2015) showed that plant WUE in subtropical regions was not significantly correlated with leaf N concentrations under phosphorus limitations but was significantly and positively correlated with leaf phosphorus concentration.

In this study, the δ^13^C values of *P. armandii* needles were negatively correlated with the soluble sugar/starch ratio, whereas those of *P. yunnanensis* needles were positively correlated with the C:N ratio. This finding indicates that the WUE of *P. armandii* is related to NSC transformation during growth. *P. armandii* responds to seasonal variation by regulating the C sources and sinks. The WUE of *P. yunnanensis* was positively correlated with N utilization efficiency. N and P are the limiting factors of plant growth and development. Their levels are closely related to photosynthetic processes and govern the production and distribution of NSC (Xie et al., 2018). The leaf N content is positively correlated with NSC fixation capacity, and P is critical for plant metabolism (McGroddy et al., 2004). Therefore, the leaf N and P contents influence the photosynthetic capacity and NSC synthesis. We analyzed the individual and interactive effects of needle N and P contents, NSC storage and conversion, and the δ^13^C values of the two tree species to determine the key factors affecting their survival and growth in different seasons. The results showed that needle C:N:P stoichiometry was significantly correlated with the NSC content, indicating that the needle N and P concentrations and the C:N and C:P ratios were related to NSC storage in the two tree species. Our results are consistent with studies of other plants (Xie et al., 2018). In this study, the N content was positively correlated with the NSC content (including soluble sugar and starch contents) and increased as NSC were stored. In contrast, the NSC content (including soluble sugar and starch contents) was negatively correlated with the P content and positively correlated with the N:P ratio (**Figure 4**). These results demonstrated that in this region limited by N, the needle P content affected the NSC storage capacity and content. There are two reasons. First, P is involved in plant metabolism and energy and protein synthesis, and the P content is closely related to the leaf N content. Second, limitations of the soil conditions may have affected the results.

## 5 Conclusion

The two pioneer species (*P. armandii* and *P. yunnanensis*) showed different ecological adaptation strategies to seasonal variation in terms of needle δ^13^C values, NSC allocation, and C:N:P stoichiometry.

The needle δ^13^C values of *P. armandii* did not change significantly over the seasons, whereas those of *P. yunnanensis* increased and decreased and were highest in summer. Sugar and starch are transformed into each other to cope with seasonal changes. The needle C:N:P stoichiometry of the two tree species showed different responses over the seasons. The needle N content was higher in spring for both tree species. A highly significant correlation occurred between needle NSC content (including soluble sugars and starch) and C:N:P stoichiometry in both tree species. In contrast, the correlations between needle δ^13^C values, C:N:P stoichiometry, and NSC content of the two tree species were not significant. The phenotypic plasticity analysis showed low indices for needle δ^13^C values of the two species, indicating that they were highly adaptive to the environment, and the degree of adaptation was higher in *P. armandii* than in *P. yunnanensis*. The seasonal variation in the soluble sugar and starch contents was more pronounced in *P. yunnanensis* than in *P. armandii*. Principal component analysis revealed that the needle NSC, starch, and P contents and the N:P ratio of the two species were important indicators of physiological strategies to cope with seasonal variation.

This study improves our understanding of the WUE strategies, C, N, and P contents, and NSC dynamic balance of the two pioneer tree species *P. armandii* and *P. yunnanensis* in medium-aged stands in subtropical China. It provides information on the trade-off between needle NSC and carbon allocation, providing a theoretical basis for forest restoration and ecosystem construction in the region. We can select suitable silvicultural species for subtropical land regions based on their water use strategy to ensure ecological adaptation under future climate change.

## Data availability statement

The original contributions presented in the study are included in the article/supplementary materials, further inquiries can be directed to the corresponding author/s.

## Author contributions

YL: Formal analysis, Writing - review & editing. JW, DZ and YB: Designed the experiments, provided critical revisions and final approval of the article. JX, JS, ZZ, and XD: Carried out the experiments and run the data. All authors also helped to write, read and approved the final manuscript.
